# Supplementary material for: A Polyamine Oxidase from Selaginella lepidophylla (SelPAO5) can Replace AtPAO5 in Arabidopsis through Converting Thermospermine to Norspermidine instead to Spermidine
Source: Plants (Basel). 2019 Apr 15;8(4):99. doi: 10.3390/plants8040099 (PMC6524367; doi:10.3390/plants8040099)
Supplement: Supplementary file 1 [file plants-08-00099-s001.pdf]

Table S1. Oligonucleotide primers used in this study.

Experiment	Name	Sequence (5`-3`)
Plasmid construction	SelPAO5_XbaI_F	AGTCTAGAATGGAGCTGAAAATATGCCAA
	SelPAO5_SmaI_F	AGCCCGGGATGGAGCTGAAAATATGCCAA
	SelPAO5_SacI_R	GGGAGCTCTTTACGATTCCAGGATTTTGTA
	SelPAO5_Sac_Mut_F	GGCCTCTATAAGGAACTCGTTGCCGAG
	SelPAO5_Sac_Mut_R	GATCTCGGCAACGAGTTCCTTATAGAG
RT-PCR	SelPAO5_ F	GATGGAGCCCACGGAAGA
	SelPAO5_ R	CGGGCCGGTGAATAAGC
	AtPAO5_ F	GTTGGGATGAACCAGAAGGA
	AtPAO5_ R	GAGGAGCCTCGGTAAGAAGA
	AtAct_ F	TCATGACCACTATCTCTTGCTTGAC
	AtAct_ R	GTTGTGGAGTAATGGGTTCTATGTG

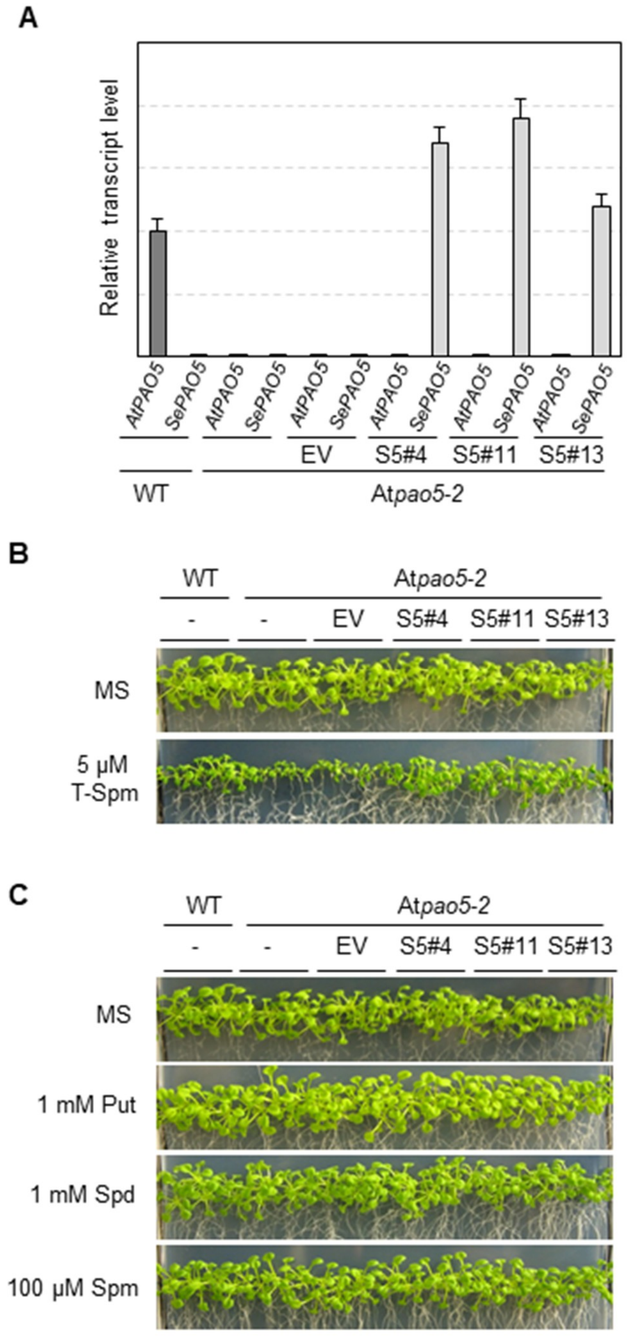

Figure S1. Recovery of T-Spm-induced growth reduction in *Atpao5-2* by complementation with *SelPAO5*. A. Relative expression levels of *AtPAO5* and *SelPAO5* compared constitutively expressed gene coding for actin (*AtActin*). The *Atpao5-2* transgenics, transformed with the empty vector (EV) or the *SelPAO5* ORF under control of the *CaMV35S* promoter, were analyzed by qRT-PCR using the appropriate gene-specific primers (Table S1). Total RNAs were prepared from the following plant samples: WT, Col-0; *Atpao5-2*; EV, *Atpao5-2* transgenic carrying the control empty binary vector pPZP2Ha3(+) [50]; S5#4, *Atpao5-2* transgenic line 4 carrying the *CaMV35S*-driven *SelPAO5*; S5#11, *Atpao5-2* transgenic line 11 carrying the *CaMV35S*-driven *SelPAO5*; and S5#13, *Atpao5-2* transgenic line 13 carrying the *CaMV35S*-driven *SelPAO5*. B. Growth phenotypes of WT, *Atpao5-2* and the *Atpao5-2* transgenics (see Figure 2A legend) on half-strength MS agar medium alone (upper), or containing 5 µM T-Spm (lower). Picture was taken 20 days after sowing. C. Growth phenotypes of WT, *Atpao5-2* and the *Atpao5-2* transgenics (see Figure 2A legend) on half-strength MS agar medium alone (top), or containing 1 mM Put (second), 1 mM Spd (third), and 100 µM Spm (bottom). Picture was taken 20 days after sowing.
